# Evaluation of the Clinical Variables Affecting Attachment Reproduction Accuracy during Clear Aligner Therapy

**DOI:** 10.3390/ma16206811

**Published:** 2023-10-23

**Authors:** Angela Mirea Bellocchio, Marco Portelli, Ludovica Ciraolo, Elia Ciancio, Angela Militi, Matteo Peditto, Serena Barbera, Riccardo Nucera

**Affiliations:** Department of Biomedical Sciences, Dentistry and Morphological and Functional Imaging, University of Messina, Via Consolare Valeria 1, 98125 Messina, Italy; mportelli@unime.it (M.P.); ludovicaciraolo@gmail.com (L.C.); ciancioelia.97@hotmail.it (E.C.); amiliti@unime.it (A.M.); mpeditto@unime.it (M.P.); serena.barbera93@gmail.com (S.B.); riccardo.nucera@gmail.com (R.N.)

**Keywords:** attachment template, attachment shape, attachment materials, attachment curing

## Abstract

Background: The purpose of this study was to evaluate some of the clinical variables that influence the accuracy of reproducing the planned attachment shape. The following clinical variables were considered: the template material, type of composite, and pressure application on the template during attachment curing. Methods: In this study, the evaluated materials for the thermoplastic transfer template construction are Erkolen 0.8 (polyethylene: PE) and Erkodur 0.8 (polyethylene terephthalate glycol—PET-G), and two types of composite resins: Enaflow (light-curing low-viscosity composite resin) and Enamel plus dentina HRI (light-curing high-viscosity composite resin). Two different light-curing lamps were used: Valo cordless color with no pressure and push light pressure (SCS). The 26 models included in the study were imported into the 3 Shape Ortho System 2022 (ver. 85.0.20 3 Shape, Denmark), and attachments were virtually placed on the dental elements of the first premolar and on both sides of the first upper molars. The accuracy of the attachment reproduction was evaluated through linear and angular evaluations against the reference model (MCAD). Three physical models were obtained: model A (MA), which was printed with attachments; model B (MB) with attachments made with a PE template; and model C (MC) with attachments made with a PET-G template. Results: The results showed statistically significant differences (*p* < 0.05) between the PE and PET-G templates with greater precision using the PET-G template. Statistically significant differences (*p* < 0.05) were found among the high-viscosity composite and low-viscosity composite with pressure curing. Conclusions: In light of the obtained data, using a PET-G template is recommended. The pressure application during composite curing reduces the reproduction accuracy with a low-viscosity composite.

## 1. Introduction

The increasing demand for aesthetic orthodontic treatment has led clinicians to embrace the use of clear aligners [1]. Align Technology©, a company based in Santa Clara, CA, USA, introduced the Invisalign^®^ system in 1998, which revolutionized the use of clear aligners in the United States [2].

Clear aligner therapy offers several advantages over traditional fixed braces, including improved aesthetics, removability, and enhanced comfort [3,4] and absence of frictional sliding mechanics force systems [5,6]. In recent years, significant investments have been made to enhance the characteristics of aligners, expanding their indications, applications, and features [7].

Moreover, the use of clear aligners in combination with miniscrews has increased the therapeutic possibility and expanded the number of eligible patients for clear aligner therapy [8].

The effectiveness and efficiency of different aligner systems depend on various factors, including the use of appropriate thermoplastic materials, optimal gingival margin design, attachment design, auxiliaries, and staging strategies [1,9,10].

Attachments, which are composite material protrusions that are bonded to the teeth, serve as force transducers, improving force exertion and aligner retention [11,12]. Different attachment shapes are designed to achieve specific treatment goals and facilitate specific tooth movements [9].

Specifically, the literature has shown that the effectiveness of orthodontic treatment with clear aligners is affected by the combination of the shape, arrangement, size, and number of attachments [3,13,14,15,16].

The presence of attachments has a positive influence on the intrusion movement and torque expression of the dental elements and increases the retention of the aligner by acting as an anchorage. Larger attachments with sharper edges have shown better performance in reproducing programmed tooth movements [11,12].

In addition, it has been shown that the use of attachments can increase the effectiveness of mesiodistal molar movement and posterior anchorage control. The attachment shape has been shown to significantly influence tooth movement, and optimized horizontal and rectangular attachments have been shown to perform better in all mesiodistal movements [11,12].

Virtually planned attachments are reproduced in vivo on a patient’s dentition using thermoplastic transfer templates.

Attachments are produced with light-cured composite resin applied using conventional adhesive procedures.

Only a limited number of studies have determined the most suitable type of composite resin for clinical applications, and none have assessed the performance of transfer template materials, which could significantly affect the effectiveness of aligners and, consequently, the final treatment outcomes.

The literature shows limited evidence on the ideal composite characteristics for attachment manufacturing in a clinical environment [17]; moreover, no study has assessed the optimal transfer template material. The precise replication of attachment shapes is critical for aligner effectiveness and to achieve treatment outcomes [14,18]. This study aims to assess some of the clinical factors that may impact the accuracy of thermoplastic template attachment reproduction, including the template material, composite type, and pressure applied during attachment curing.

## 2. Materials and Methods

The research protocol was reviewed and approved by the Ethics Committee of the University of Messina (prot. no. 33-2020). This study was conducted according to the guidelines of the Declaration of Helsinki. The materials used in this study are reported in Table 1. Two types of composite resins were selected: Enaflow (Micerium Spa, Avegno, Genova, Italy) and Enamel plus dentina HRI (Micerium Spa, Avegno, Genova, Italy). Two different materials were used for the construction of the thermoplastic transfer template: polyethylene (PE) 0.8 mm trays (Erkolen, Erkodent, Erich Kopp Gmbh, Pfalsgrafenweier, Germany) and polyethylene terephthalate glycol (PET-G) (Erkodur, Erkodent, Erich Kopp Gmbh, Pfalsgrafenweier, Germany).

### 2.1. Material Characteristics and Curing Protocols Used in the Creation of the Attachment

The characteristics of the composite resins used are as follows:

The EnaFlow is composed of a monomeric matrix of diurethandimethacrylate and 1,4-butanedioldimetha-crylate, and this composition provides the material with excellent flowability, allowing for the easy adaptation and placement of the material during attachment reproduction. The total filler content is 33% by weight, the inorganic fillers (0.04–3.0 µm) are light-cured, and the fillers contribute to the strength and durability of the material, ensuring that the attachments remain intact throughout treatment. Enaflow is a light-curing composite resin that has a low viscosity; this characteristic allows it to flow easily into attachment molds and achieve the accurate reproduction of attachments. This characteristic is critical to ensure optimal attachment between attachments and clear aligners, facilitating tooth movement and aligner retention [19].

Additional features are the low-viscosity, biocompatible, and Bis-GMA-free composite.

The Enamel plus dentin HRI consists of urethane dimethacrylate and tricyclodecane dimethanol dimethacrylate. The total filler content is 74 wt% (60% by volume); the particle size of the silicon dioxide has a high dispersion and measures 0.005–0.05 µm, while the particle size of the glassy filler measures 0.2–3.0 µm. This composition provides the material with high viscosity, good compactness, and strength, making it suitable for attachment reproduction. In addition, the Enamel plus dentin HR, due to its composition, has a high viscosity and good compactness and strength. This characteristic allows for better control during attachment placement, ensuring accurate reproduction and adaptation to the tooth surface [20,21].

The characteristics of the transfer template materials used are as follows:

PE is a soft and elastic material. This characteristic allows for the template to be easily adapted and shaped to the tooth’s surface, ensuring a precise fit. The flexibility of PE masks allows for comfortable placement and removal during the transfer process.

The thickness of PE templates used for the transfer attachment is typically 0.8 mm. This thickness provides adequate rigidity and stability to the template, allowing for the accurate reproduction of the shape and position of the attachment. An additional characteristic that affects its effectiveness as a template is its viscoelasticity, i.e., it can deform under stress but returns to its original shape after the stress is removed. This property allows for the template to conform closely to the tooth’s surface during attachment transfer, ensuring the accurate reproduction of attachment details.

PET-G exhibits viscoelasticity and hardness characteristics. It has a higher viscoelasticity than PE, which allows for a good fit and contour to the tooth’s surface. In addition, the hardness of PET-G imparts stability and rigidity to the template, ensuring the accurate reproduction of the shape and position of the attachment.

PET-G templates used for transfer attachment typically have a thickness of 0.8 mm. This thickness ensures adequate stability and durability of the template during the transfer process.

PET-G is known to have greater strength and durability compared to PE. It can withstand the forces and stresses encountered during attachment transfer, ensuring the reliable and consistent reproduction of the attachments.

Template thermoforming process:

Material template preparation (PE or PET-G): Initially, a template sheet with a thickness of 0.8 mm is carefully selected.

Dental model preparation: Before commencing thermoforming, a dental model is prepared; it is printed in resin using a 3D printer. The dental model must be accurate and free of debris or residues.

Material heating: The template sheet is then heated in a thermoforming oven at a specific temperature, usually ranging between 150 °C and 200 °C, depending on the material specifications.

Thermoforming: Once the desired temperature is reached, the PET-G sheet is placed over the prepared dental model. Using controlled pressure or a thermoforming machine, the material is shaped to perfectly fit the dental arches and desired orthodontic attachments.

Cooling: After thermoforming, the template is gradually cooled. Cooling stabilizes the template in its final form. During this process, it is important to prevent distortions or deformations in the template.

Quality control: The thermoformed template is carefully inspected to ensure it fits accurately on the dental model, with all details clearly visible. An adequate thermoforming process ensures a precise fit of the template to the model and thus a good fit of the template and, consequently, a more precise reproduction of the shape of the attachments to be reproduced.

Attachments were polymerized using two light-curing lamps and two different protocols: the UV Grand Valo lamp (Ultradent, 505 West Ultradent Drive, South Jordan, UT 84095, USA) was used without a pressure protocol, and the “Push and light” lamp (La Compagnia Ortodontica, Via Montefiore, 1207, 47521 Cesena FC, Italy) was used with an applied pressure protocol.

### 2.2. Sample Selection and Operative Procedures

Twenty-six digital maxillary models of patients who were referred to the outpatient orthodontic clinic were selected.

The models were selected by the archives of the clinic according to the following inclusion criteria: models of Caucasian subjects aged between 14 and 35 years, first molar dental class, first canine dental class, any agenesis, any dental anomalies, intact permanent dentition (excluding third molars), absence of severe crowing and severe rotation, and little index ≤ 3 mm.

The models included in the study were imported in the Ortho Analyzer software (3 Shape, Copenhagen K, Denmark), and attachments were virtually placed on the dental elements on the first premolars and first upper molars on both sides. All attachments created were rectangular with beveled edges and were placed in the center of the clinical crown with a long axis of the attachment parallel to the vertical axis of the dental element.

This process generated a new CAD file Model for each patient, named MCAD. The model was designed with applied attachments, and both of the models (with and without attachments) were exported and printed. For each patient, the following models were produced with a 3D printing process using a Liquid Crystal Precision 1.5 3D printer (Photocentrinc Inc., Avondale, AZ, USA) with Daylight Precision Model White Resin (Photocentrinc Inc., Avondale, AZ, USA): two models without attachment and one model with attachment.

The MCAD with printed attachment was used to thermoform two templates of different materials (PE and PET-G) with a thickness of 0.8 mm.

The obtained thermoplastic attachment templates were used for attachment construction on the printed models with various combinations to test all the considered variables.

**Figure 1:**
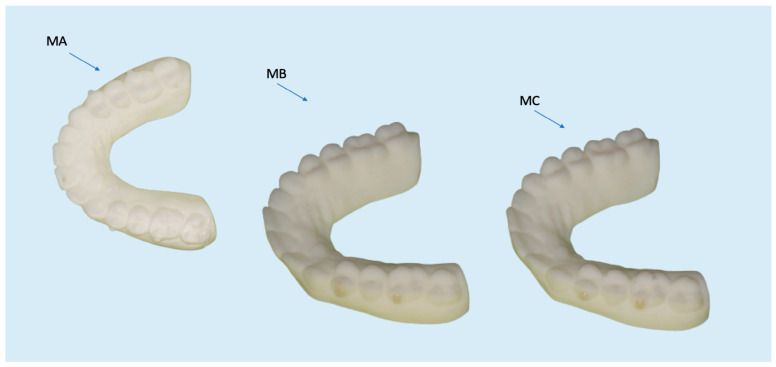
Model A (MA) printed with attachments, Model B (MB) with attachments made with PE template, and Model C (MC) with attachments made using PET-G template.

With this procedure, 3 physical models were obtained: Model A (MA) printed with attachments, Model B (MB) attachments made with composite and PE template, and Model C (MC) attachments made with composite and PET-G template (Figure 1). Attachments were applied to premolars and molars on both sides. The attachments on the premolars were created with Enamel Plus, while on the molars. they were created with Enaflow. The attachments were then cured on the left hemi-maxillary without the pressure technique, and they were cured on the right hemi-maxillary with the pressure technique. MA was scanned at time T0 immediately after printing, and MB and MC were digitized after making the attachments. Moreover, MA was scanned a second time one week after printing (at time T1). All of the models’ digitalizations were executed using Medit I-500 intra-oral scanner (Medit, Seoul, Republic of Korea)Lastly, the four digital models (MA-T0, MB, MC, and MA-T1) were compared to the master model (MCAD) exported from the 3 Shape software (3Shape Ortho System 2022 (3 Shape, Copenhagen K, Denmark)).

Geomagic Control X software (https://oqton.com/geomagic-controlx/) was used to perform digital model superimposition.

### 2.3. 3D Analysis

**Figure 2:**
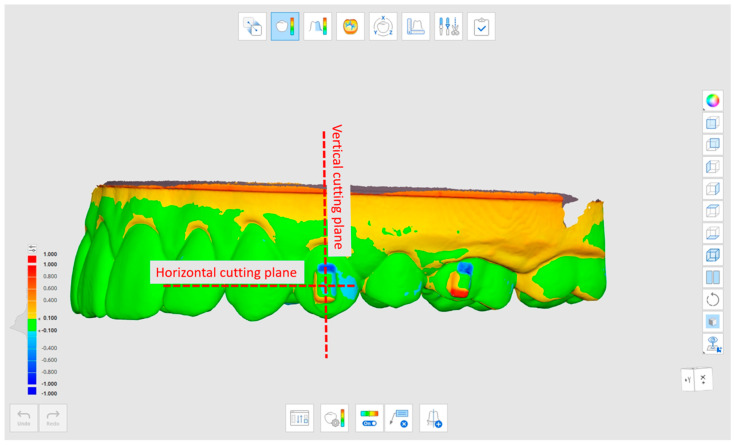
Reference plans used to assess discrepancies. The vertical cutting plane (Ver) and the horizontal cutting plane.

By using Medit Compare, the discrepancies between the superimposed models were evaluated with specific outcomes that were measured in vertical cut planes (according to the attachment long axis) and in horizontal cut planes (according to the attachment short axis) (Figure 2).

**Figure 3:**
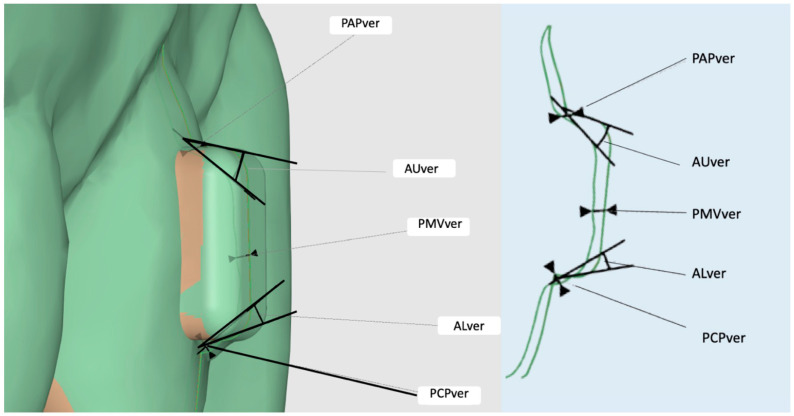
Outcomes evaluated in the vertical cutting plane.

In the vertical cutting plane, the models were compared by assessing the following outcomes:

The maximum discrepancy between the two most apico-palatal attachment points of the two models (Point–Apico-Palatal–vertical: PAPver) (Figure 3).

The maximum discrepancy between the two most coronal–palatal points of the attachments of the two models (Point–Coronal–Palatal–vertical: PCPver) (Figure 3).

The maximum discrepancy between the two middle-vestibular points of the attachments of the two models (Point–Middle–Vestibular–vertical: PMVver) (Figure 3).

The angle measured between the upper horizontal attachment profiles of the two models (Angle–Upper–vertical: AUver) (Figure 3).

The angle measured between the lower horizontal attachment profiles of the two models (Angle–Lower–vertical: ALver) (Figure 3).

**Figure 4:**
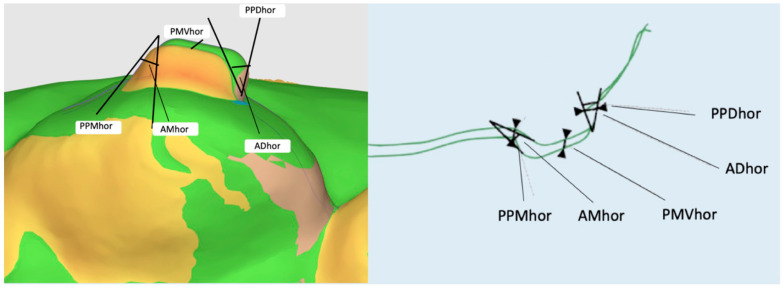
Outcomes evaluated in the horizontal cutting plane.

In the horizontal cut plane, the models were compared by assessing the following outcomes:

The maximum discrepancy between the two most mesial–palatal attachment points of the two models (Point–Palatal–Mesial–horizontal: PPMhor) (Figure 4).

The maximum discrepancy between the two most disto-palatal attachment points of the two models (Point–Palatal–Distal–horizontal: PPDhor) (Figure 4).

The maximum discrepancy between the two middle-vestibular attachment points of the two models (Point–Middle–Vestibular–horizontal: PMVhor) (Figure 4).

The angle formed between the upper approximal attachment profiles of the two models (Angle–Mesial–horizontal: AMhor) (Figure 4).

The angle formed between the approximate lower attachment profiles of the two models (Angle–Distal–horizontal: ADhor) (Figure 4).

All evaluated outcomes are reported and described in Table 2.

For each attachment, to consider it in its entirety and shape, a total of 10 outcomes were evaluated: 5 outcomes on the vertical cut plane and 5 outcomes on the horizontal cut plane. Four attachments were assessed for each model, and comparisons were made between four different models: MCAD versus MA-T0, MCAD versus MA-T1, MCAD versus MB, and MCAD versus MC.

A total of 160 outcomes were assessed for each patient.

### 2.4. Statistical Analysis

Descriptive and inferential statistics were performed using SPSS statistical software (version 25.0; IBM Corporation, Armonk, NY, USA).

The significance levels were set at *p* < 0.05. The distribution of the data was assessed with Shapiro–Wilk tests and the homogeneity of variance was assessed with Levene’s test. Inferential statistics were performed using parametric ANOVA multiple comparison tests.

#### 2.4.1. Methodological Error

To assess the methodological error related to the scanning and model overlapping processes, the outcomes of the MA-T0 and MA-T1 models were compared.

The paired *t*-test and intraclass correlation coefficient (ICC) were used to assess intra-operator reliability. The magnitude of random error was assessed using Dahlberg’s formula. No significant differences (*p* < 0.05) were noted between the two assessments; all measurements were highly reliable, with the ICC ranging from 0.85 to 0.96.

The random error ranged from 0.08 to 0.12 mm.

#### 2.4.2. Power Analysis

Preliminary power analysis was performed on the first five patients enrolled according to the methodology described above.

The analysis was conducted while considering the preliminary mean values of the outcome and assessing the relative linear discrepancy of PAPver MCAD vs. MA-T0 and MCAD vs. MB, and the common standard deviation was also assessed.

The analysis was performed with a power of 80%, and the significance level was set at 0.05.

The analysis showed a sample size of 23 cases. Enrollment was set at 26 patients to minimize the risk of false negatives.

## 3. Results

The results of the descriptive statistics are reported in Table 3 and Table 4.

The descriptive statistics revealed that the vertical cut plane analysis showed a greater discrepancy for the attachments that were produced with PE templates compared to the attachments that were produced with PET-G templates. AUver showed a maximum mean angle value of 35.41°, and PMver revealed a maximum mean linear discrepancy value of 0.62 mm (Table 3).

The inferential statistics considered the mean differences in the linear discrepancy in the vertical planes of the PAPver, PCPver, and PMVver outcomes, and in the horizontal planes of the PPMhor, PPDhor, and PMVhor outcomes about the considered variables of the present study.

To compare PE and PET-G, flow composite was considered the gold standard in attachment reproduction because of its widespread use among clinicians.

The results showed significant differences (*p* < 0.05) between the PE and PET-G templates in the horizontal and vertical cut plane evaluations for all of the evaluated outcomes obtained with curing pressure (Table 5).

The attachments that were created without curing pressure showed significant differences related to the transfer template material (PE vs. PET-G) for all of the considered outcomes except for the most apical outcome (PAPver) and middle outcome (PMVver) (Table 5).

The results showed statistically significant differences when comparing the composite paste and composite flow in the vertical and horizontal cut planes for all of the outcomes assessed with pressure (Table 5)

The angular discrepancies were also evaluated, and the results showed statistically significant differences between the evaluations on the vertical cut plane between the most apical portion of the attachment (AUver) and the most coronal portion of the attachment (ALver). The horizontal cut plane between the most mesial (AMhor) and the most distal attachments (ADhor) was compared (Table 6).

The results showed statistically significant differences for all of the variables analyzed in the vertical plane when there was pressure; on the other hand, no statistically significant differences were present in the absence of pressure when paste composite and flow composite were used (Table 7).

## 4. Discussion

Attachments used in combination with clear aligners are essentially designed to improve tooth movement during orthodontic treatment. If attachments are not properly designed or positioned, they may not be able to exert the necessary force to move teeth to the desired planned position. Another aspect that potentially affects the efficacy of clear aligner treatment and its clinical outcome is the precision of attachment shape reproduction. Numerous studies evaluate the effectiveness of different attachment shapes in reproducing the desired tooth movement [3,11,12,15]. However, few studies in the literature evaluated the role of the composite type in the accuracy of reproducing the programmed attachment shape in vivo [17,22]. No study has assessed the accuracy of clear templates in reproducing the planned attachment shape. This study, for the first time, evaluates the effects of some specific variables that affect the final attachment shape reproduction, specifically the material template, composite characteristics, and pressure application during composite curing. To the best of our knowledge, this is the first study that performed a comprehensive evaluation of the above-mentioned clinical variables.

In order to design a clinical-oriented statistical analysis strategy, PET-G and flow composite were considered as the gold standards in the attachment reproduction. This choice was performed due to the widespread use among clinicians of the protocol combining the use of PET-G and flow composite. Consequently, a univariate ANOVA was performed only for the two above-mentioned characteristics.

The results of the univariate ANOVA analysis showed that the use of different template materials can generate significant discrepancies between the planned and final attachment shapes.

Specifically, polyethylene terephthalate glycol (PET-G) is significantly more accurate than polyethylene (PE) in reproducing a planned attachment shape (*p* < 0.05).

This result can be related to the materials’ characteristics. PET-G is stiffer compared to PE and it is able, during the thermoforming process of the template, to reproduce, with greater accuracy, the anatomical characteristics of the prototyped model and, ultimately, of the programmed attachment shape [23,24].

These aspects can improve precise fitting between the template and denture, thus improving attachment reproduction accuracy.

However, the higher stiffness of PET-G can also cause some disadvantages such as immediate attachment loss for spontaneous debonding during template removal. In this regard, some authors compared PET-G and PE templates and reported that PE showed less immediate attachment debonding compared to the PET-G template. The better performance of PE may result from the greater elasticity of this template material; this characteristic could be responsible for the observed reduction in the immediate attachment debonding during template removal [25].

To assess which parts of the attachment are reproduced most accurately, a specific analysis was conducted. On a vertical cutting plane, the angular discrepancy between the horizontal profile portions (both occlusal and gingival) of the attachment produced with different procedures was measured and compared (Figure 3).

The comparison of the occlusal discrepancy angle (ALver) and the gingival discrepancy angle (AUver) revealed that the greatest discrepancy was registered from the gingival discrepancy angle (AUver).

This datum indicates that the aligner template reproduces a significantly better occlusal portion of the attachment compared to the gingival portion.

This result can be explained by the evidence that the clear template has a better fit on the occlusal portions of the model compared to the gingival portion. Our results are in accordance with the study of Park SY and co-workers that evaluated the median gap of thermoformed clear aligners using micro-CT and a spectrophotometer. The authors’ findings showed that the median gap at the gingival level was always greater compared to the occlusal level [26].

In this study, the best fitting on the occlusal portion can be explained by the characteristics of the thermoforming process. In fact, during the thermoforming procedures, the template is less adherent to the gingival portion of the model for the presence of model undercuts; this aspect reduces template fitting and prevents optimal attachment reproduction [27,28].

Regarding the attachment material, the paste composite and flow composite showed similar performances, with registered non-significant differences (*p* > 0.05), when no pressure was applied on the template during curing. On the contrary, the paste composite and flow composite showed significant differences when pressure was applied during curing. In particular, with pressure application, the paste composite was shown to be the best material for reproducing the attachment shape (Table 3 and Table 4).

These results can be explained by considering the viscosity and surface tension properties of the tested materials. The greater fluidity of the flow composite is related to a lower presence of inorganic filler and the presence of low-viscosity resin monomers [11].

In a previous study [17], the authors have shown that resins with lower viscosities are less reliable in reproducing the shape of the attachment, reporting that fluid resin materials “overflow under pressure when placing the template”; these findings are confirmed by the results of the present investigation.

Other authors [14,29] compared three different materials used to create the attachment, i.e., flowable composite, conservative packable composite, and orthodontic composite; these authors concluded that all three materials are accurate in reproducing the shape of the attachment. The results of the previously mentioned study are confirmed by the results of this study.

However, no study has evaluated attachment reproduction accuracy with pressure application during curing. In this experimental condition, this study found significant differences between low- and high-viscosity composites, showing the best reproduction results when pressure application was associated with a high-viscosity composite.

Therefore, when a cure pressure protocol is applied, the first choice should be to use a high-viscosity paste packable composite to achieve optimal attachment reproduction. The use of a low-viscosity flow composite could be related to composite overflows around the attachment, causing the need for excess composite removal in the same appointment. This could determine an increase in chair time during the attachment placement appointment.

Lin S and co-workers previously compared flowable and packable composites in terms of preparation times and attachment damage rates, finding shorter preparation times for flowable composite resins and no significant difference between these two types of composite materials in terms of the attachment damage rates during the first year [30].

An overall evaluation of all of the material properties involved in the attachment creation is essential in order to select the most appropriate materials according to the clinician’s needs.

This study provides new information related to the ideal characteristics of materials used for attachment implementation. However, the present investigation has limitations related to the experimental design of the study.

Further clinical studies are needed to validate the above-mentioned experimental results and to assess the effects that precise attachment reproduction has on ideal tooth movement achievement.

## 5. Conclusions

The study data showed a greater shape accuracy of attachments made with a PET-G transfer template compared to a PE transfer template. However, PET-G also had disadvantages such as immediate attachment debonding. This study found that the clear template reproduced the occlusal portion of the attachment better than the gingival portion, which was possibly due to a better fit on the occlusal surface during thermoforming. When pressure was applied during curing, the paste composite showed the best performance in reproducing the attachment shape. Further clinical studies are needed to validate these findings and assess the impact on desired tooth movement.

## Figures and Tables

**Table 1:** Materials used in the study.

Materials	Template	Curing Lamps
Enaflow (Micerium)	Erkolen 0.8 Erkodent (PE)	UV Grand Valo lamp
Enamel plus HRI (Micerium)	Erkodur 0.8 Erkodent (PET-G)	Push light pressure (SCS)

**Table 2:** Descriptions of outcomes considered.

Outcome	Description
PAPver	Maximum discrepancy between the two most apico-palatal points of the attachments of the two models
PCPver	Maximum discrepancy between the two most coronal–palatal points of the attachments of the two models
PMVver	Maximum discrepancy between the two middle-vestibular points of the attachments of the two models
AUver	Angle formed between the upper horizontal profiles of the attachments of the two models
ALver	Angle formed between the lower horizontal profiles of the attachments of the two models
PPMhor	Maximum discrepancy between the two most mesio-vestibular points of the attachments of the two models
PPDhor	Maximum discrepancy between the two most disto-vestibular points of the attachments of the two models
PMVhor	Maximum discrepancy between the two middle-vestibular points of the attachments of the two models
AMhor	Angle formed between the upper approximal profiles of the attachments of the two models
ADhor	Angle formed between the approximate lower profiles of the attachments of the two models

**Table 3:** Descriptive statistics in the outcome of the evaluated vertical cutting plane.

Materials	Curing Lamps	Template	PAPver			PCPver			PMVver			AUver			ALver		
			Mean ± SD (mm)	Min (mm)	Max (mm)	Mean ± SD (mm)	Min (mm)	Max (mm)	Mean ± SD (mm)	Min (mm)	Max (mm)	Mean ± SD (°)	Min (°)	Max (°)	Mean ± SD (°)	Min (°)	Max (°)
Composite flow	No pressure	CAD-PE	0.33 ± 0.04	0.23	0.41	0.33 ± 0.17	0.09	0.63	0.62 ± 0.23	0.28	0.94	32.3 ± 3.4	26.19	36.74	19.36 ± 1.03	14.74	29.8
Composite flow	No pressure	CAD-PEG	0.30 ± 0.14	0.15	0.67	0.27 ± 0.07	0.13	0.4	0.41 ± 0.09	0.12	0.42	23.17 ± 5.03	19.2	27.31	6.1 ± 1.03	4.77	8.07
Composite paste	No pressure	CAD-PE	0.34 ± 0.05	0.22	0.41	0.25 ± 0.15	0.08	0.34	0.29 ± 0.08	0.26	0.53	34.59 ± 2.22	16.4	37.9	14.8 ± 2.3	10.3	18.21
Composite paste	No pressure	CAD-PEG	0.3± 0.14	0.18	0.73	0.23 ± 0.29	0.09	0.65	0.28 ± 0.09	0.05	0.37	26.57 ± 0.93	24.83	28.92	5.48 ± 3.4	3.4	9.3
Composite flow	Pressure	CAD-PE	0.26 ± 0.04	0.14	0.34	0.3 ± 0.1	0.18	0.53	0.42 ± 0.32	0.29	0.85	29.95 ± 0.7	25.72	32.2	17.93 ± 0.75	14.49	22.37
Composite flow	Pressure	CAD-PEG	0.24 ± 0.10	0.13	0.44	0.15 ± 0.07	0.05	0.31	0.16 ± 0.04	0.08	0.56	21.23 ± 3.2	19	23.25	6.4 ± 1.05	4.33	8.02
Composite paste	Pressure	CAD-PE	0.21 ± 0.05	0.13	0.33	0.21 ± 0.26	0.06	0.92	0.36 ± 0.1	0.23	0.63	35.41 ± 1.2	34.09	37.01	13.23 ± 0.68	12.26	14.73
Composite paste	Pressure	CAD-PEG	0.20 ± 0.07	0.08	0.37	0.19 ± 0.15	0.11	0.75	0.13 ± 0.07	0.04	0.31	25.8 ± 0.96	24.28	27.8	3.93 ± 2.8	2.87	5.2

**Table 4:** Descriptive statistics in the outcome of the evaluated horizontal cutting plane.

Materials	Curing Lamps	Template	PMMhor			PDDhor			PMVhor			AMhor			ADhor		
			Mean ± SD (mm)	Min (mm)	Max (mm)	Mean ± SD (mm)	Min (mm)	Max (mm)	Mean ± SD (mm)	Min (mm)	Max (mm)	Mean ± SD (°)	Min (°)	Max (°)	Mean ± SD (°)	Min (°)	Max (°)
Composite flow	No pressure	CAD-PE	0.36 ± 0.27	0.27	0.29	0.33 ± 0.03	0.13	0.39	0.28 ± 0.18	0.11	0.6	13.29 ± 5.02	8.19	18.8	22.57 ± 4.66	15.68	27.29
Composite flow	No pressure	CAD-PEG	0.29 ± 0.11	0.11	0.25	0.27 ± 0.07	0.19	0.48	0.19 ± 0.04	0.07	0.25	13.22 ± 0.15	13.13	13.7	22.4 ± 2.87	21.44	25.4
Composite paste	No pressure	CAD-PE	0.17 ± 0.07	0.07	0.22	0.25 ± 0.04	0.04	0.37	0.18 ± 0.05	0.14	0.3	17.28 ± 2.02	13.16	22.5	23.88 ± 5.81	11.9	28.04
Composite paste	No pressure	CAD-PEG	0.15 ± 0.07	0.07	0.2	0.23 ± 0.04	0.13	0.29	0.17 ± 0.04	0.12	0.37	16.3 ± 5.01	14.24	21.8	22.98 ± 4.58	19.7	26.4
Composite flow	Pressure	CAD-PE	0.31 ± 0.21	0.21	0.23	0.3 ± 0.06	0.16	0.45	0.3 ± 0.07	0.19	0.49	22.4 ± 2.98	16.36	26	14.22 ± 0.98	12.12	16.4
Composite flow	Pressure	CAD-PEG	0.22 ± 0.15	0.15	0.19	0.15 ± 0.05	0.11	0.33	0.19 ± 0.04	0.13	0.29	21.3 ± 0.99	24.18	22.8	13.24 ± 2.99	11.18	15.8
Composite paste	Pressure	CAD-PE	0.16 ± 0.13	0.13	0.17	0.21 ± 0.06	0.09	0.35	0.19 ± 0.04	0.09	0.26	22.72 ± 4.6	18.08	27.05	18.68 ± 7.56	15.01	26.08
Composite paste	Pressure	CAD-PEG	0.14 ± 0.12	0.12	0.15	0.19 ± 0.05	0.07	0.27	0.16 ± 0.05	0.01	0.31	18.77 ± 2.53	16.5	21.15	17.77 ± 1.54	16.18	19.7

**Table 5:** Inferential statistics and multiple comparison (univariate ANOVA). a means significant differences with *p* < 0.05 were detected; b means no significant differences were detected.

	PAPver	PCPver	PMVver	AUver	ALver	PMMhor	PDDhor	PMVhor	AMhor	ADhor
PE vs. PET-G pressure	0.03 ^a^	0.01 ^a^	0.02 ^a^	0.04 ^a^	0.05 ^a^	0.03 ^a^	0.02 ^a^	0.01 ^a^	0.04 ^a^	0.05 ^a^
PE vs. PET-G no pressure	0.9 ^b^	0.04 ^a^	0.5 ^b^	0.03 ^a^	0.02 ^a^	0.01 ^a^	0.03 ^a^	0.02 ^a^	0.24 ^b^	0.04 ^a^
Composite paste vs. composito flow pressure	0.04 ^a^	0.02 ^a^	0.16 ^b^	0.01 ^a^	0.04 ^a^	0.02 ^a^	0.01 ^a^	0.05 ^a^	0.04 ^a^	0.02 ^a^
Composite paste vs. composito flow no pressure	0.07 ^b^	0.17 ^b^	0.01 ^a^	0.02 ^a^	0.03 ^a^	0.04 ^a^	0.14 ^b^	0.03 ^a^	0.01 ^a^	0.19 ^b^

**Table 6:** Inferential statistics and multiple comparisons for angular evaluation (univariate ANOVA). a means significant differences with *p* < 0.05 were detected. b means no significant differences were detected.

	Composite Paste vs. Composite Flow		PE vs. PETG	
Total Ver pressure	0.04 ^a^		0.01 ^a^	
Total Ver no pressure	0.22 ^b^		0.27 ^b^	
Total Hor pressure	0.03 ^a^		0.02 ^a^	
Total Hor no pressure	0.17 ^b^		0.31 ^b^	
	**Composite Paste**	**Composite Flow**	**PE**	**PET-G**
AUver vs. ALver pressure	0.04 ^a^	0.05 ^a^	0.03 ^a^	0.02 ^a^
AUver vs. ALver no pressure	0.03 ^a^	0.02 ^a^	0.01 ^a^	0.03 ^a^
AMhor vs. ADhor pressure	0.01 ^a^	0.04 ^a^	0.02 ^a^	0.05 ^a^
AMhor vs. ADhor no pressure	0.02 ^a^	0.03 ^a^	0.04 ^a^	0.01 ^a^

**Table 7:** Inferential statistics and multiple comparisons for total outcomes evaluated (univariate ANOVA). a means significant differences with *p* < 0.05 were detected; b means no significant differences were detected.

	Composite Paste vs. Composite Flow	PE vs. PET-G
Total Ver pressure	0.05 ^a^	0.03 ^a^
Total Ver no pressure	0.14 ^b^	0.33 ^b^
Total Hor pressure	0.02 ^a^	0.01 ^a^
Total Hor no pressure	0.29 ^b^	0.25 ^b^

## Data Availability

The authors will provide raw data upon reasonable request.
